# Multifunctional Halloysite-Glutathione Nanocomposite for Solar CO2 Conversion and Pollutant Sensing

**DOI:** 10.1021/acsanm.5c05781

**Published:** 2026-02-14

**Authors:** Erika Saccullo, Angelo Ferlazzo, Giusy Dativo, Roberto Fiorenza, Giulia Sambataro, Elena Bruno, Antonino Gulino, Antonio Rescifina, Vincenzo Patamia, Giuseppe Floresta

**Affiliations:** † Department of Drug and Health Sciences, 599012 University of Catania, Viale Andrea Doria 6, 95125 Catania, Italy; ‡ Department of Biomedical and Biotechnological Sciences (Biometec), University of Catania, Via Santa Sofia 97, 95123 Catania, Italy; § Department of Chemical Sciences, University of Catania, Viale Andrea Doria 6, 95125 Catania, Italy; ∥ Department of Physics and Astronomy “Ettore Majorana”, University of Catania, via S. Sofia 64, 95123 Catania, Italy; ⊥ CNR-IMM, Via Santa Sofia 64, 95123 Catania, Italy

**Keywords:** HNT, nanotube, chelating agent, bisphenol A, CO_2_ photoconversion, electrochemical nanosensor

## Abstract

This study presents a highly sustainable nanocomposite platform for dual applications in both photocatalysis and sensing. The nanomaterial is synthesized via a green process using natural, readily available components, including halloysite nanotubes (HNTs), glutathione (GSH), and xanthopterin, utilizing copper ions (Cu^1+^) as the catalytically active species. The synthesis, which exclusively uses green solvents like tetrahydropyran (THP) and water, involves functionalizing HNTs with (3-aminopropyl)­triethoxysilane (APTES), followed by the attachment of GSH as a chelating agent for Cu^1+^. Finally, xanthopterin is loaded to act as a light-harvesting antenna. Fourier-transform infrared (FT-IR) spectroscopy and thermogravimetric analysis (TGA) confirmed the successful functionalization and composition. Scanning electron microscopy (SEM) and energy-dispersive X-ray (EDX) analysis validated the material’s morphology and elemental composition. The resulting nanocomposite, HNT-NH_2_-GSH-Cu^1+^-X, demonstrated a remarkable synergistic effect, achieving a CO_2_ conversion of 42.2% and a high (86.1%) CH_4_ selectivity. Furthermore, the HNT-GSH-Cu^2+^ nanocomposite exhibited excellent electrochemical sensing capabilities for bisphenol A, with a low limit of detection (LOD) of 0.022 μM and a high sensitivity of 5.098 μA μM^–1^·cm^–2^. The work successfully demonstrates the creation of a sustainable, multifunctional nanomaterial that addresses critical environmental challenges by combining efficient solar fuel production with highly sensitive pollutant detection.

## Graphical abstract

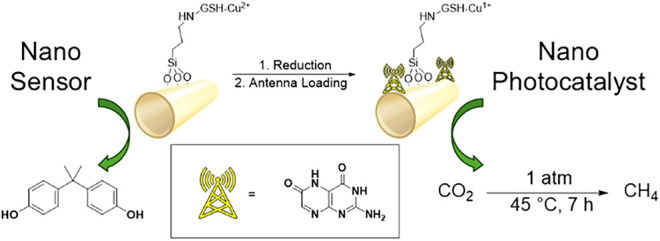

## 1 Introduction

Developing new multifunctional nanomaterials is a critical challenge in addressing global issues such as sustainability and environmental protection.^1,2^ Systems capable of performing multiple tasks simultaneously, such as the photoconversion of CO_2_ to methane and the detection of pollutants like bisphenol A, offer integrated, efficient solutions to both energy and environmental crises. Indeed, the possibility of producing solar fuels, such as methane, from CO_2_ aligns with current calls for the development of a sustainable, nonfossil-fuel-based carbon economy. These solar fuels could be used for the synthesis of strategic chemicals or, if used in combustion processes, the emitted CO_2_ could be balanced with the starting material used to produce the same solar fuels, enabling high-value-added products from a greenhouse gas with zero-carbon-emission processes. The importance of this work is underscored by the need to develop such systems sustainably, using green solvents and materials derived from renewable natural resources.

In this context, the use of sustainable nanomaterials, such as natural polysaccharides (cellulose, chitosan, and alginic acid) and natural clays, is transforming catalysis and sensing. These nanomaterials offer eco-friendly alternatives to synthetic compounds due to their abundance, biodegradability, and biocompatibility.^3,4^ Their matrices are often integrated with chelating agents that bind metal ions, creating hybrid systems with unique functionalities.^5–7^ In catalysis, polysaccharides and halloysite nanotubes (HNTs) serve as versatile supports for immobilizing metal catalysts^8^ (e.g., palladium or copper nanoparticles) via chelating groups.^9^ This approach not only enhances catalyst stability but also facilitates its recovery and reuse, significantly reducing costs and environmental impact

In sensing, the ability to chelate metal ions enables the development of highly selective and sensitive sensors.^10–12^ Their functionality is based on the change in the material’s optical or electrochemical properties when an analyte binds to the chelating agent.^13,14^

The choice between using HNTs and glutathione (GSH) is particularly significant. HNTs, natural clay minerals with tubular structures, provide a versatile scaffold that supports catalytically active agents.^9,15–17^ Thanks to their high surface area and stability, HNTs are also ideal for immobilizing receptors in electrochemical sensors, thereby improving their selectivity and sensitivity toward specific analytes such as organic pollutants, metal ions, and biomarkers.^18–20^

GSH, a natural tripeptide, acts as a reducing and stabilizing agent for nanoparticles, a crucial aspect of their catalytic activity.^21–23^ In the field of sensing, GSH’s thiol group offers highly specific recognition sites with a strong affinity for heavy metal ions (e.g., mercury or cadmium) and other pollutants.^24,25^ This property enables the creation of sensors with exceptional sensitivity for the detection of environmental contaminants.

By combining these two materials via a green synthesis route, this work aims to create a new, multifunctional nanoplatform that is not only effective for critical applications but also prepared in accordance with environmental sustainability principles. Despite the low copper content, this composite can function as an electrochemical nanosensor for bisphenol A. In the presence of an antenna of natural origin, it is also capable of converting CO_2_ into CH_4_ photochemically. Indeed, rather than the most commonly used and critical noble metals, the choice of copper as the photocatalytically active species is a more sustainable and green approach to producing solar fuels from CO_2_, as copper is economical and not critical. Furthermore, particularly the cuprous ion, Cu (I), as reported in the literature, favors the selective CO_2_ reduction^9,26–28^ into methane.

## 2 Materials

### 2.1 General Information

All chemicals were purchased from Merck and VWR. Halloysite (HNT, cat. no. 685445), CuCl_2_ (powder, 99%). Data for this article, including SEM-EDS, TGA, IR, and Electrochemical data are available at zenodo.org at https://zenodo.org/records/17276779?token=eyJhbGciOiJIUzUxMiJ9.eyJpZCI6IjNhMzdmYmE3LTRhNjUtNDM4MS1iZTlhLTczOTg4MmQyZmRhZiIsImRhdGEiOnt9LCJyYW5kb20iOiJkODA5ZmRkMTRhNjQyMzE3ZDAxYWY3MDFkOTgyNzAzYiJ9.D3pB5oTMRSCgzMARKuKdXDsVUI1X7UXSWiZceoeNYcd7kqd1h7MYVlyDJiUdFX4PK0X0KNiFjoTXdSeC5yx4hA (10.5281/zenodo.17276778).

### 2.2 Synthesis of Materials

#### 2.2.1 Synthesis of HNT-NH_2_

Two mL of APTES [(3-aminopropyl)­triethoxysilane] and 10 mL of THP were added to 400 mg of HNT and stirred at 65 °C for 12 h. At the end of the reaction, the crude reaction mixture was centrifuged, and the solid was repeatedly washed with THP (3 × 5 mL) and then centrifuged to remove impurities. Finally, the solid was dried in an oven at 70 °C for 24 h to obtain 413 mg of HNT-NH_2_.

#### 2.2.2 Synthesis of HNT-NH_2_-GSH

150 mg of GSH were suspended in 12 mL of THP under a nitrogen atmosphere, to which 112 mg of EDC [1-ethyl-3­(3-(dimethylamino)­propyl) carbodiimide] were added, then left to stir for 10 min under a nitrogen atmosphere. At the end of this time, 200 mg of HNT-NH_2_ and 5 mL of THP were added, and the resulting mixture was stirred for 48 h at room temperature under a nitrogen atmosphere. The crude reaction mixture was then centrifuged (at rt and 5000 rpm), and the solid obtained was washed with deionized water (3 × 5 mL) and finally dried under vacuum to obtain 220 mg of HNT-NH_2_-GSH.

#### 2.2.3 Synthesis of HNT-NH_2_-GSH-Cu^2+^

To a dispersion of HNT-NH_2_-GSH (100 mg) in H_2_O (5 mL) was added CuCl_2_ (50 mg).^29^ The resulting mixture was stirred at 55 °C overnight. The reaction mixture was then centrifuged and washed several times with deionized H_2_O to remove excess uncomplexed metal ions. The supernatant was removed, and the solid was dried under a vacuum to give 104 mg of product.

#### 2.2.4 Synthesis of HNT-NH_2_-GSH-Cu^1+^

To a dispersion of HNT-NH_2_-GSH (100 mg) in H_2_O (5 mL) was added CuCl_2_ (50 mg).^29^ The resulting mixture was stirred at 55 °C overnight. To reduce Cu­(II) to Cu­(I) in the resulting HNT-NH_2_-GSH-Cu^2+^ product, the sample was suspended in 6 mL of a 0.5 M ascorbic acid solution, which was then stirred for 3 h at room temperature. Finally, the suspension was centrifuged at 5300 rpm for 10 min, then washed three times with water. The supernatant was removed, and the solid was dried under vacuum to give 102 mg of product.

#### 2.2.5 Procedure for Loading of Xanthopterin

100 mg portion of HNT-NH_2_-GSH-Cu^1+^ was mixed with xanthopterin (10 mg) in an agate mortar. The physical mixture was then repeatedly ground until a homogeneous, opaque yellow final product (HNT-NH_2_-GSH-Cu^1+^-X) was obtained. To evaluate loading efficiency, we performed the same experiment as reported in our previous work.^28^ A 10:1 (w/w) mixture of HNT-NH_2_-GSH-Cu^1+^ (25 mg) and xanthopterin (2.5 mg) was dispersed in 1 mL of DMSO and stirred continuously at room temperature overnight. The resulting suspension was centrifuged at 5300 rpm for 20 min, and the supernatant was analyzed by UV (Jasco V-730 spectrophotometer and quartz cuvettes with an optical path length of 1 cm).

### 2.3 Experimental Setup

#### 2.3.1 General Procedure for the Solar Photocatalytic CO_2_ Reduction

The photocatalytic tests were conducted at atmospheric pressure using a cylindrical batch Pyrex reactor filled with 0.1 g of sample, which was irradiated for 7 h with a solar lamp (Osram Ultra Vitalux 300 W, irradiance of 10.7 mW/cm^2^). A mixture of CO_2_ (99.999%) and H_2_ gas (evolved by an HK Hydrogen generator, purity 99.9996%) was flowed into the photoreactor to saturate the sample surface with the reagent molecules. With a mass flow controller, the H_2_/CO_2_ mixture was tuned to favor CO_2_ methanation (H_2_/CO_2_ molar ratio 4:1).^30^

The reaction products (under our experimental conditions, only CO and CH_4_) were examined using an Agilent 8860 gas chromatograph (Carboxen column, TCD detector) and a Trace GC instrument (Porapak Q column, FID detector), both of which were adequately calibrated. The measurements were repeated three times (3% experimental error).

The CO_2_ conversion values were calculated with eq 1

1 $${\mathrm{CO}}_{2}\;\mathrm{conversion}\;\%:\left(\frac{{a\mathrm{rea}}_{{\mathrm{peak CO}}_{2}\;\mathrm{in}}-{a\mathrm{rea}}_{{\mathrm{peak CO}}_{2}\;\mathrm{out}}\times \left(\frac{{a\mathrm{rea}}_{\mathrm{peak standard in}}}{{a\mathrm{rea}}_{\mathrm{peak standard out}}}\right)}{{a\mathrm{rea}}_{{\mathrm{peakCO}}_{2}\;\mathrm{in}}}\right)\times 100$$

Moreover, the mass balance method was also applied, considering eq 2

2 $${\mathrm{CO}}_{2}\;\mathrm{conversion}\;\%:\left(\frac{{\mathrm{area}}_{{\mathrm{peak CO}}_{2}\;\mathrm{out}}}{{\mathrm{area}}_{\mathrm{peak products out}}+{\mathrm{area}}_{{\mathrm{peak CO}}_{2}\;\mathrm{out}}}\right)\times 100$$

The two methods were in agreement (±5%, 95% reproducibility).

The selectivity of the CO and CH_4_ products on an electron basis was evaluated with eqs 3 and 4 ^31^

3 $${\mathrm{CH}}_{4}\;\mathrm{selectivity}\;\%:\left(\frac{8N_{CH_{4}}}{\left(8N_{CH_{4}}+2N_{\mathrm{CO}}\right)}\right)\times 100$$

4 $$\mathrm{CO}\;\mathrm{selectivity}\;\%:\left(\frac{2N_{\mathrm{CO}}}{\left(8N_{CH_{4}}+2N_{\mathrm{CO}}\right)}\right)\times 100$$

where N_CH_4__ and N_CO_ are the production rates of CH_4_ and CO in μmol/g_catalyst_·h_irradiation_, and the coefficients 8 and 2 are used to account for the electrons involved in the photocatalytic reduction reactions to form CH_4_ and CO from the CO_2_ using H_2_ as the electron donor and proton source, and considering the reactions ([Disp-formula eq5]) and ([Disp-formula eq6]).^31–33^

a $${\mathrm{CO}}_{2}+2H^{+}+2e^{-}\to \mathrm{CO}+H_{2}O$$

b $${\mathrm{CO}}_{2}+8H^{+}+8e^{-}\to {\mathrm{CH}}_{4}+2H_{2}O$$

The CH_4_ yield (%) was calculated with eq 7

5 $${\mathrm{CH}}_{4}\;\mathrm{yield}\;\%:\left(\frac{{\mathrm{CH}}_{4}\;\mathrm{selectvity}\;\%\times {\mathrm{CO}}_{2}\;\mathrm{conversion}\;\%}{100}\right)$$

#### 2.3.2 Electrochemical Measurements

Cyclovoltammetry (CV), linear sweep voltammetry (LSV), and differential pulse voltammetry (DPV) measurements were performed using a DropSens μStat-i 400s potentiostat/galvanostat equipped with Dropview 8400 software. A 0.1 M phosphate-buffered saline (PBS) solution (pH 7.4) was used for electrochemical measurements. CV tests were performed at a scan rate of 50 mV/s in the −0.2–1.0 V potential range. LSV tests were conducted using a 0.1 M PBS solution at a scan rate of 50 mV s^–1^ within a 0–1.0 V potential range. Measurements were made using a commercial reference Screen-Printed Carbon Electrode (SPCE), from the Methrom DropSens company, and a working SPCE modified with HNT-GSH-Cu (HNT-GSH-Cu/SPCE) by depositing on it (0.125 cm^2^) 25 μL of a suspension of HNT-GSH-Cu^2+^ (5 mg in 1 mL of distilled water). DPV tests were conducted using an optimized potential step (*E*_step_) of 0.03 V, a potential pulse (*E*_puls_) of 0.09 V, and a time pulse (*T*_pul_) of 200 ms, with a scan rate of 40 mV/s in the 0.0–1.0 V potential range. Afterward, all measurements were repeated after the appropriate volumes (0.025–2.5 L) of a 10 mM bisphenol A solution were added to the 0.1 M PBS electrolyte solution (pH 7.4). The resulting sensors were dried at room temperature under a nitrogen atmosphere for 24 h.^12,34^ The sensor sensitivity (*S*) was always calculated (eq 8) as the ratio between the slope (*m*) of the calibration line and the geometric surface area (*A*) of the modified SPCE electrode (0.125 cm^2^).^35^ The Limit of Detection (LOD) was calculated by the relationship between the standard deviation (SD) of the blank (solutions without any bisphenol A) and the slope (*a*) obtained from the calibration curve as follows (eq 9)^36^

6 $$S=\frac{m}{A}$$

7 $$\mathrm{LOD}=3.3\times \frac{\mathrm{SD}}{a}$$

### 2.4 Characterization

#### 2.4.1 Infrared Spectroscopy

FTIR-ATR analyses were conducted using an FTIR Agilent Cary 630 instrument equipped with an ATR sampling module. Thin films of the samples were applied to the ATR crystal and pressed gently. The results were derived from 512 scans acquired in the 4000–500 cm^–1^ range with a resolution of 2 cm^–1^ at room temperature.

#### 2.4.2 Thermogravimetric Analysis

Thermal gravimetric analysis (TGA) was performed under 1 atm of prepurified nitrogen at a heating rate of 10 °C/min, in the temperature range of 50–900 °C. The instrument used is the PerkinElmer TGA 4000.

#### 2.4.3 Evaluation of the Mean Particle Size and Polydispersity Index

To evaluate the mean particle size (Z-ave) and polydispersity index (PdI) of HNT, HNT-NH_2_, HNT-NH_2_-GSH, and HNT-NH_2_-GSH-Cu^2+^, they were solubilized/suspended in water (1 mg/mL) and analyzed using Photon Correlation Spectroscopy (PCS) with a Zetasizer Nano S90 instrument (Malvern Instruments, Malvern, U.K.). The instrument was set to a detection angle of 90 °C and a 4 mW He–Ne laser operating at 633 nm with the temperature set to 25 °C. Three sets of measurements were used in the sample analysis, and the mean size ± standard deviation (SD) was reported.

#### 2.4.4 SEM-EDS

The synthesized material’s morphology was analyzed by scanning electron microscopy using a Dual Beam Focused ion beam Versa 3D LoVac Dual Beam in secondary electron mode using a 5 keV electron beam. The samples were also analyzed in situ by using energy-dispersive X-ray spectroscopy (EDS) with a 20 keV electron beam. Before analysis, all samples were sputtered with 5 nm of Au to ensure proper conductivity during measurements.

#### 2.4.5 UV-DRS

The ultraviolet visible-diffusion (UV–vis DRS) spectra were acquired using a Jasco V-670 instrument equipped with an integrating sphere, using BaSO_4_ as reference.

#### 2.4.6 Textural Properties

The textural properties of the samples were evaluated using N_2_ physisorption measurements at −196 °C with a Micromeritics ASAP 2020 instrument after outgassing the samples overnight at 80 °C. The surface area of the samples was determined by the Brunauer–Emmett–Teller (BET) method. The pore size distribution was obtained using the Barrett–Joyner–Halenda (BJH) model (from the desorption curves).

#### 2.4.7 X-ray Photoelectron

X-ray photoelectron spectra (XPS) were measured at a 45° takeoff angle relative to the surface sample holder, with a PHI 5000 Versa Probe II system (ULVAC-PHI, INC., base pressure of the main chamber 1 × 10^–8^ Pa).^37,38^ Samples were dispersed on a Si substrate and excited with the monochromatized Al Kα X-ray radiation using a pass energy of 5.85 eV. The instrumental energy resolution was ≤0.5 eV. The XPS peak intensities were obtained after Shirley’s background removal.^37,38^ Spectra calibration was achieved by fixing the Ag 3d_5/2_ peak of a clean sample at 368.3 eV.^39^ The atomic concentration analysis was performed by considering the relevant atomic sensitivity factors. XP spectra were fitted with XPSPEAK4.1 software using Gaussian envelopes after background subtraction. This process involves data refinement utilizing the method of least-squares, which was continued until the highest possible correlation between the experimental spectrum and the theoretical profile was achieved. The residual or agreement factor *R*, defined by *R* = [Σ (*F*_obs_ – *F*_calc_)^2^/Σ (*F*_obs_)^2^]^1/2^, after minimization of the function Σ (*F*_obs_ – *F*_calc_)^2^, converged to the value of 0.03.

## 3 Results and Discussion

### 3.1 Synthesis

This work aims to utilize a sustainable, eco-biocompatible nanomaterial based on readily prepared natural materials for a sustainable application, such as the photoconversion of CO_2_ into CH_4_. The entire synthetic process is further enhanced by the use of green solvents such as tetrahydropyran (THP)^40^ and water, which are used in all synthetic steps (Scheme 1). The synthesis begins with the insertion of an amino termination using (3-aminopropyl)­triethoxysilane (APTES) and the hydroxyl groups on the surface of HNT, with THP serving as the solvent. We then decided to conjugate GSH, a naturally occurring tripeptide, to serve as a chelator of Cu,^41,42^ which is helpful for the photoconversion of CO_2_. This was then reduced to Cu^1+^ in water in the presence of ascorbic acid. Finally, mimicking nature, an antenna, xanthopterin, also of natural origin, was loaded to increase the number of photons captured and enhance the photoconversion yield. This approach has previously been used by our research group^28^ to improve the conversion yield of CO_2_ to CH_4_ by inserting xanthopterin, which, as wasps do,^43^ captures more photons and enhances catalysis.

**Scheme 1:**
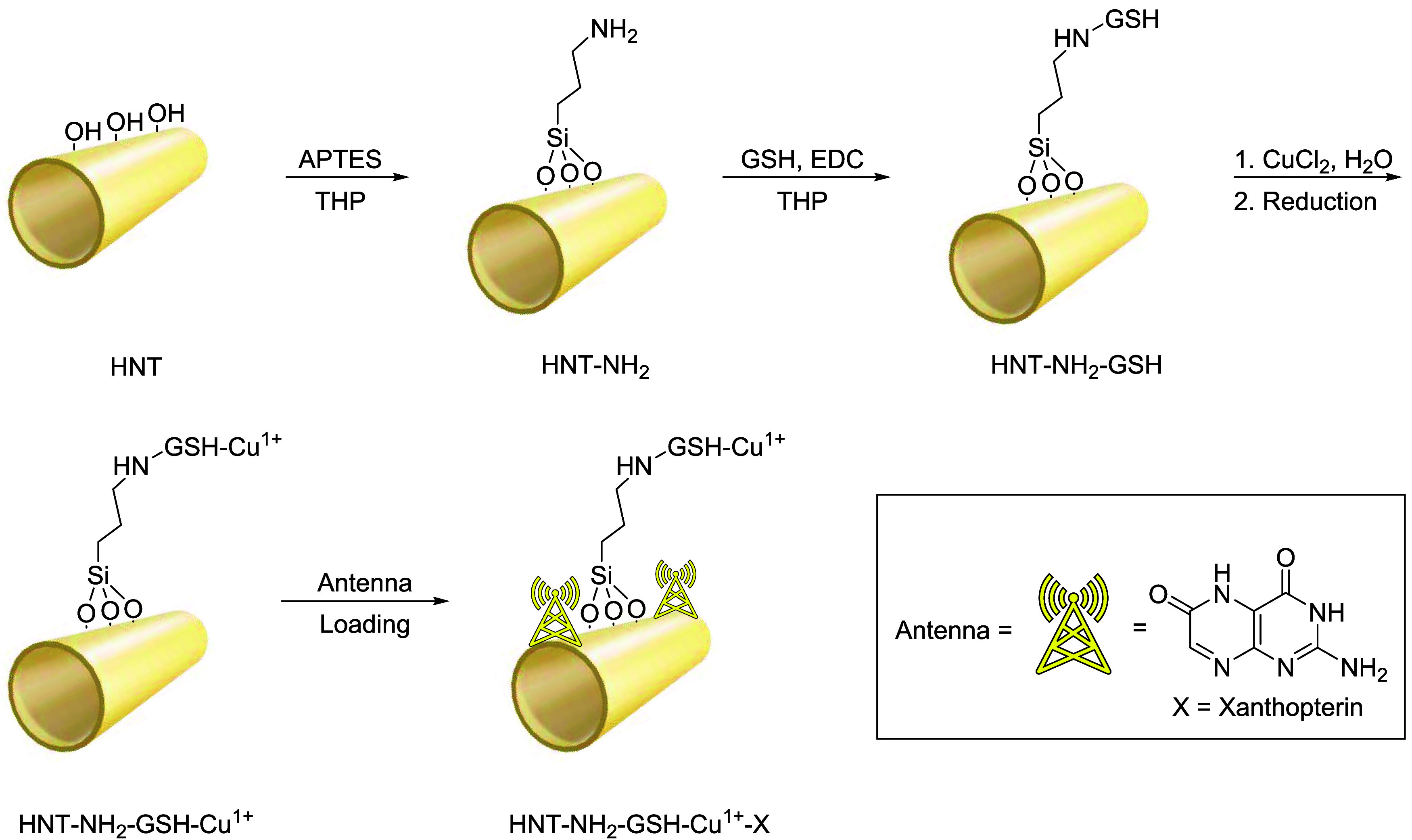
Preparation of HNT-NH_2_-GSH-Cu^1+^-X

### 3.2 Characterizations

Successful functionalization was confirmed by FT-IR analysis. Figure 1 shows the FT-IR spectra of HNT, HNT-NH_2_, and HNT-NH_2_-GSH, demonstrating successful functionalization. The bands associated with the OH groups are visible in the HNT spectrum (black line): the Al–O–OH vibration is responsible for the peak at 907 cm^–1^, while the stretching vibrations of the Al–OH groups are responsible for the bands at 3696 and 3624 cm^–1^. In addition, a prominent O–Si–O peak is observed at approximately 1000 cm^–1^, while the apical Si–O stretching mode is responsible for the peak at 749 cm^–1^.^5,15^ In addition to these, there are signals related to functionalization with APTES (red line), namely the vibrational bands at 1500–1551 cm^–1^, attributed to N–H deformation, and 1400 cm^–1^ due to the bending of C–H bonds in the carbon chain.^44^ The blue line in Figure 1 shows the spectrum of the HNT-NH_2_-GSH composite, in which an increase in the intensity of the above-mentioned signals in the 1500–1400 cm^–1^ range can be seen, and new signals in the 3000–2840 cm^–1^ region related to the stretching of C–H bonds typical of the GSH peptide chain and at 1390 cm^–1^ related to the C–N bond.^23,44^

**Figure 1:**
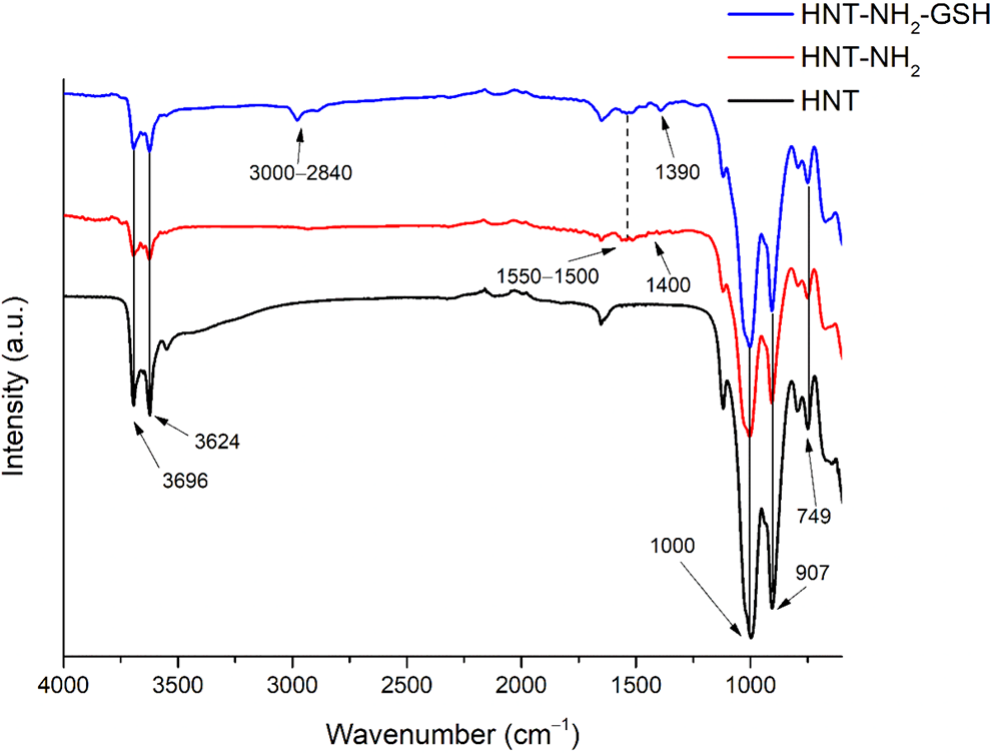
Stacked FT-IR spectra of HNT (black line), HNT-NH_2_ (red line), and HNT-NH_2_-GSH (blue line).

To verify the thermal properties, degree of functionalization (*%f*), and metal content of the synthesized nanocomposites, thermogravimetric analyses were performed.^9,28^ The overlapping thermograms of the various composites HNT (black line), HNT-NH_2_ (red line), HNT-NH_2_-GSH (blue line), HNT-NH_2_-GSH-Cu^1+^ (purple line), and HNT-NH_2_-GSH-Cu^2+^ (green line) are shown in Figure 2.

**Figure 2:**
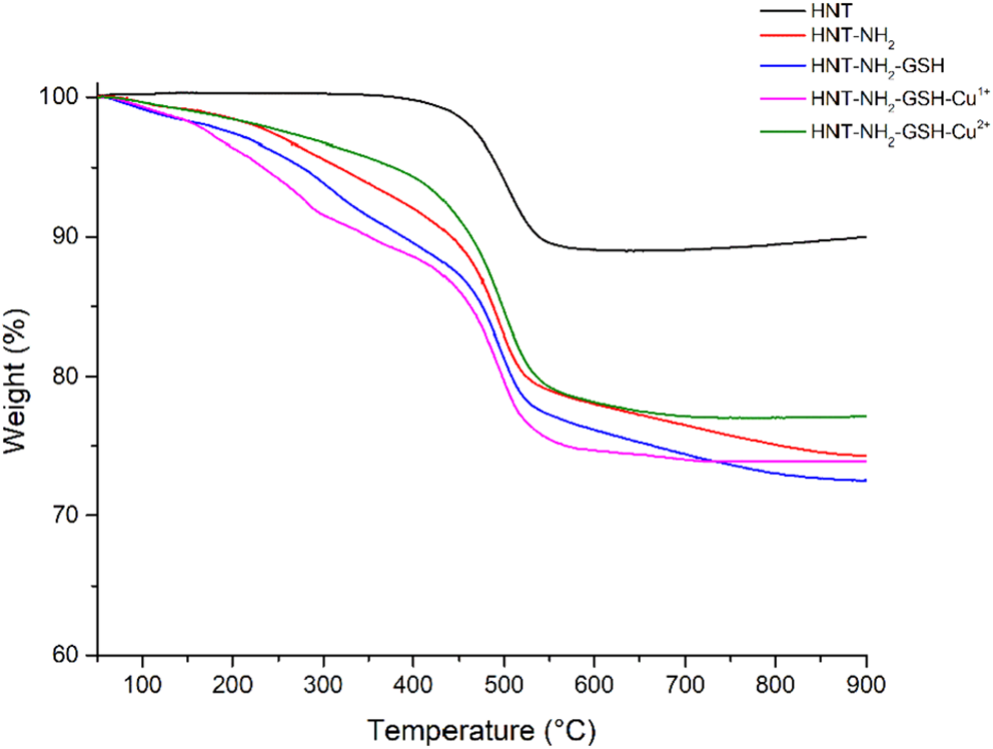
Thermogravimetric curves of HNT (black line), HNT-NH_2_ (red line), HNT-NH_2_-GSH (blue line), HNT-NH_2_-GSH-Cu^1+^ (purple line), and HNT-NH_2_-GSH-Cu^2+^ (green line).

Table 1 shows the weight loss percentages of pristine HNT, HNT-NH_2_, HNT-NH_2_-GSH, and HNT-NH_2_-GSH-Cu^1+^. The degree of functionalization (*%f*) for the composite was calculated as reported in literature^9,28^ by considering the mass loss between 150 and 550 °C. In particular, we note that % *f* for HNT-NH_2_ is approximately 6%, while for HNT-NH_2_-GSH it is approximately 7%. Finally, it was also possible to calculate the amount of metal by calculating the difference between the residue of HNT-NH-GSH and HNT-NH_2_-GSH-Cu^2+^ or HNT-NH_2_-GSH-Cu^1+^, which is equal to 4.9% and 1.5 wt %, respectively.

**Table 1:** Mass Loss Percentages of Pristine HNT, HNT-NH_2_, HNT-NH_2_-GSH, HNT-NH_2_-GSH-Cu^2+^, and HNT-NH_2_-GSH-Cu^1+^

	mass loss%					
sample	*T* < 150 °C	150 °C <*T* < 350 °C	350 °C <*T* < 550 °C	550 °C <*T* < 900 °C	residue%	amount of metal%
HNT	1.10	1.80	12.30	1.10	83.70	—
HNT-NH_2_	0.95	5.37	14.74	4.57	74.37	—
HNT-NH_2_ -GSH	1.78	7.20	14.06	4.55	72.41	—
HNT-NH_2_ -GSH-Cu^1+^	1.96	7.15	14.48	2.52	73.89	1.5
HNT-NH_2_ -GSH-Cu^2+^	0.94	3.40	16.39	1.96	77.31	4.9

The sizes of the aqueous suspensions of HNT, HNT-NH_2_, HNT-NH_2_-GSH, and HNT-NH_2_-GSH-Cu^2+^ were analyzed by using PCS (Table 2). The results confirmed that HNT has dimensions in the micrometer range (1103 nm), consistent with reports in the literature.^45^ Functionalization with APTES (NH_2_) and subsequently with GSH increased the size to 2299 and 4134 nm, respectively. The presence of copper decreases the material’s size by making the complex that forms more soluble and thus limiting the formation of aggregates, highlighting GSH chelation (2211 nm).^18,46^

**Table 2:** Mean Size (Z-ave) and Polydispersity Index (PdI) of HNT, HNT-NH_2_, HNT-NH_2_-GSH, HNT-NH_2_-GSH-Cu^2+^

sample	Z-ave (nm) ± SD	PDI ± SD
HNT	1103 ± 131.2	0.598 ± 0.20
HNT-NH_2_	2299 ± 188.1	0.458 ± 0.05
HNTK-NH_2_-GSH	4134 ± 628.6	0.863 ± 0.12
HNTK-NH_2_-GSH-Cu^2+^	2211 ± 199.5	0.330 ± 0.10

To calculate the amount of xanthopterin loaded by the nanosystem, the absorption spectra of 0.025 mg/mL xanthopterin in DMSO before and after loading were compared (Figure S1). From the comparison, it is clear that the absorption peak at 387 nm, characteristic of xanthopterin in DMSO, is completely depleted after the solution is shaken in the presence of the HNT-NH_2_-GSH-Cu^1+^-X nanocomposite. The disappearance of this band indicates that almost all xanthopterin has been effectively sequestered by the nanoconjugate, achieving a loading efficiency of 99%.^28^

The morphology of HNT-NH_2_-GSH-Cu^1+^-X was analyzed by using SEM measurements (Figure 3). The sample shows the typical tubular structure of halloysite nanotubes.^9,17,28^

**Figure 3:**
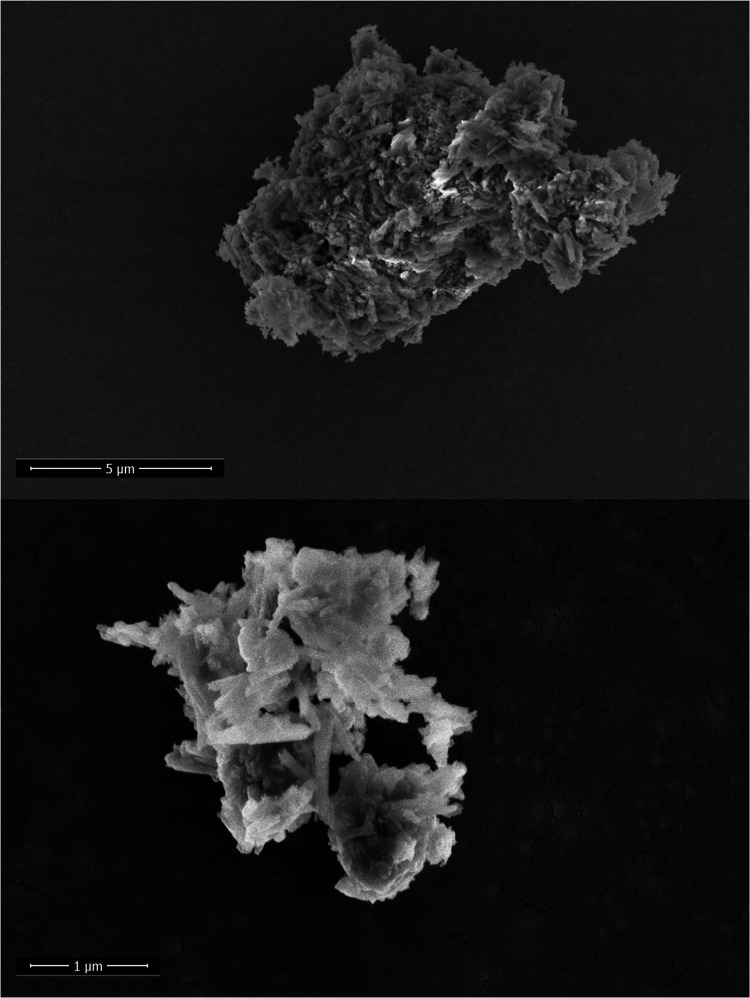
SEM micrograph of HNT-NH_2_-GSH-Cu^1+^-X.

In conjunction with the SEM analyses, EDS measurements were performed. The element map shown in Figure 4 confirms the presence of all elements, such as C, N, S, and Cu, following the functionalization and loading of xanthopterin, together with those typical of HNT, namely, Si, Al, and O, as expected.

**Figure 4:**
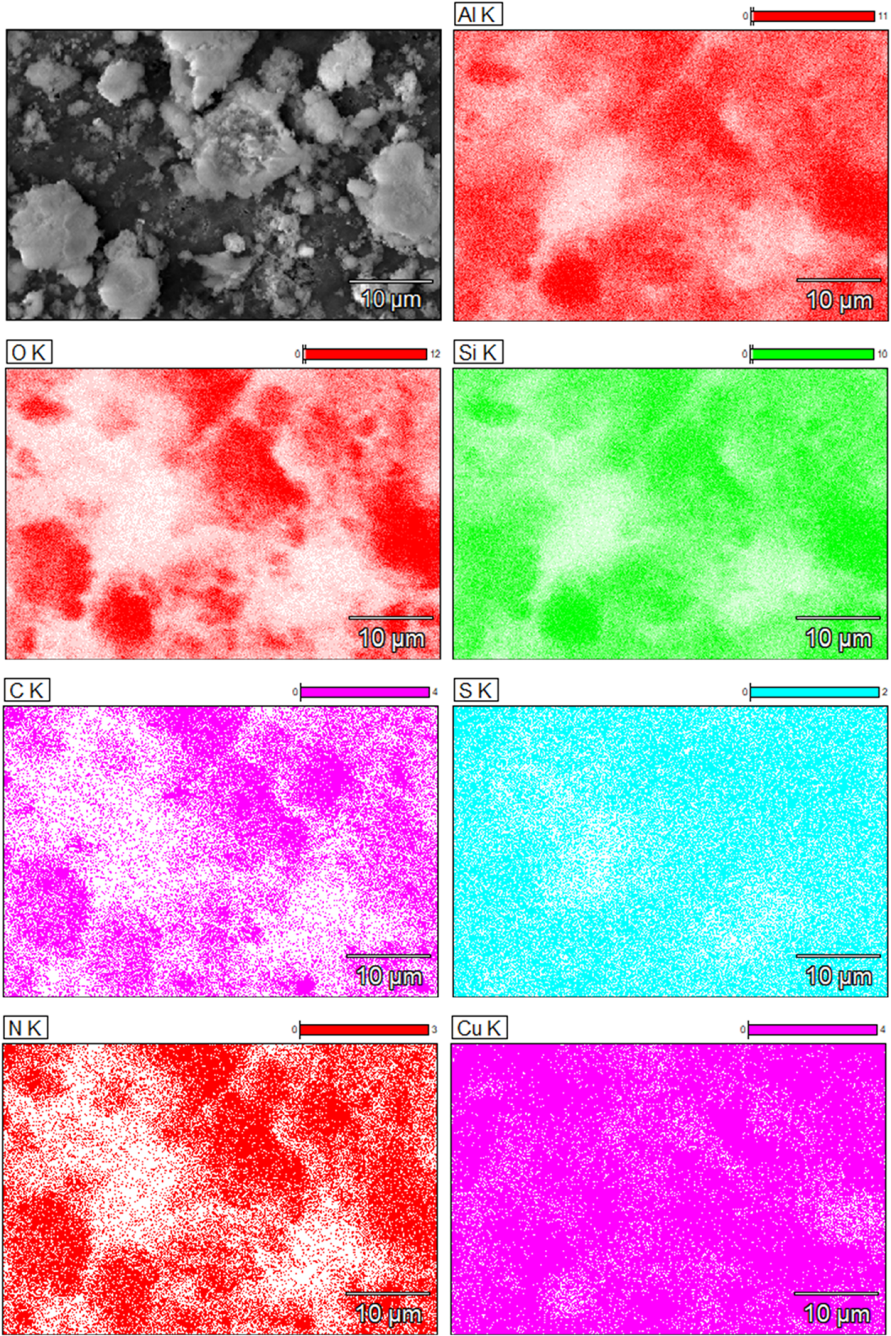
Energy-dispersive X-ray (EDS) mapping analysis of HNT-NH_2_-GSH-Cu^1+^.

Figure S2 shows the XPS survey spectrum of HNT-NH_2_-GSH-Cu^1+^-X. Peaks corresponding to C 1s, O 1s, Si 2p, Al 2p, S 2p, and N 1s are evident, together with some additional peaks of other states of the same elements. High-resolution XPS of HNT-NH_2_-GSH-Cu^1+^-X was also performed in the C 1s, O 1s, Si 2p, Al 2p, S 2p, N 1s, and Cu 2p binding energy regions (Figure S3a–f). In particular, Figure S3e shows a peak at 99.7 eV with an evident shoulder at 100.2 eV due to the Si 2p spin–orbit components of the silicon substrate on which HNT-NH_2_-GSH-Cu^1+^-X was deposited for the XPS measurements. In addition, the broad band centered at about 103 eV is due to different Si–O states in halloysite (101.9 eV) and APTES (103.8 eV), as well as some SiO_*x*_ (102.8 eV) of the substrate surface on which the sample was deposited for XPS measurement (101.9 eV).^47,48^ The Al 2p band is at 74.0 eV, typical of Al_2_O_3_ states.^49^ Surface functionalization with GSH is demonstrated by the presence of N 1s (398.9 eV) and S 2p (168.4 eV) states in an N/S = 0.9 ratio.

This latter spectrum, aside from the main peak, shows a lower-energy component at 165.6 eV due to the S–Cu­(I) states.^50^ The presence of a higher-energy component (168.6 eV) is likely due to the metal-driven Haber–Weiss reaction, which forms oxidized sulfur species that lead to sulfonic acid.^51^

Figure 5 shows the XP spectrum of HNT-NH_2_-GSH-Cu^1+^-X in the Cu 2p binding energy region. The Cu 2p_3/2,1/2_ main spin–orbit components are located at 932.1–951.9 eV with some lower components at 935.0–954.8 eV (spin–orbit coupling 19.8 eV). These energy values, together with the presence of a small satellite peak at 942.4 eV above the Cu 2p_3/2_ signal, confirm the presence of Cu­(I) (relative intensity 89.9%) and a small quantity of Cu­(II) (intensity relative 9.1%).^52,53^ Also, XPS atomic concentration analysis suggests an S/Cu surface ratio of 3.1.

**Figure 5:**
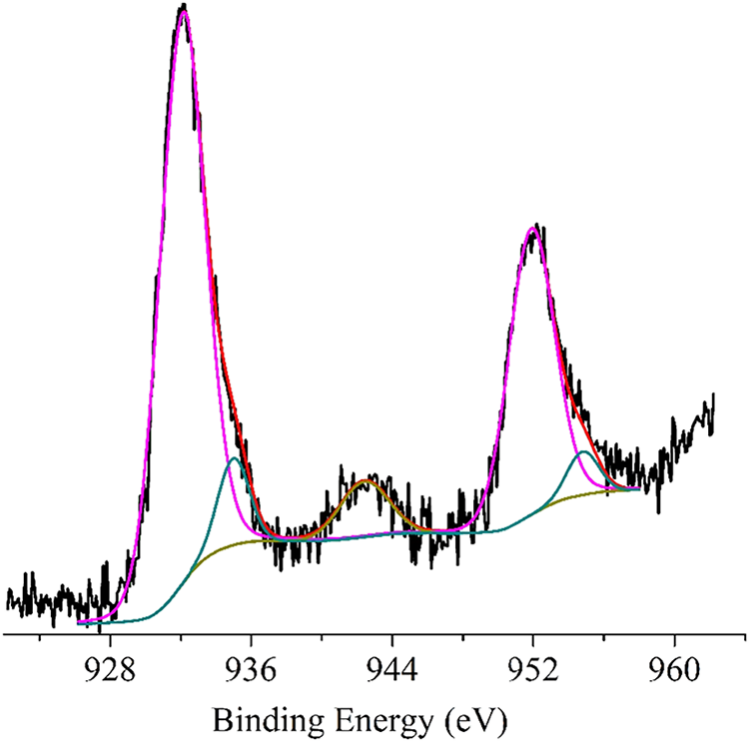
Al Kα excited XPS of the HNT-NH_2_-GSH-Cu^1+^-X sample in the Cu 2p binding energy region: the magenta, dark cyan, and dark yellow lines refer to the 932.1, 935.0, and 942.4 eV Gaussian components, respectively. The blue line represents the background, and the red line superimposed on the experimental black profile refers to the sum of the Gaussian components.

### 3.3 Photocatalytic Activity

The synthesized nanomaterials were tested for solar CO_2_ photocatalytic methanation. In our experimental conditions, only CO and CH_4_ were found as the main products of the reaction. In Table 3, the experimental data obtained are reported, comparing the different nanocomposites. As expected, the sample without the Cu^1+^ ions (bare HNT, HNT-X, HNT-NH_2_, and HNT-NH_2_-GSH) gave negligible activity (CO_2_ conversion ≤5%) due to the absence of photocatalytically active sites (as the cuprous ions) able to promote the CO_2_ reduction. As reported, the cuprous ions provided the necessary electrons to drive the reaction toward the selective formation of methane.^31,32,54^ The presence of only xanthopterin and HNT was not sufficient to photoconvert carbon dioxide.

**Table 3:** Screening of Photocatalysts Used for the Photocatalytic CO_2_ Reduction^a^

entry	sample	CO_2_ conversion after 7 h of solar radiation (simulated)	CH_4_ selectivity	CO selectivity	CH_4_ yield	CH_4_ formation rate (μmol/g_cat_·h)	CO formation rate (μmol/g_cat_·h)
1	HNT	—	—	—	—	—	—
2	HNT-Cu^1+^	7.6%	57.1%	41.9%	4.3%	1.44	1.06
3	HNT-X	3.0%	—	—	—	—	—
4	HNT-NH_2_	—	—	—	—	—	—
5	HNT-NH_2_ -GSH	5.1%	12.2%	86.8%	0.6%	0.21	1.47
6	HNT-NH_2_ -GSH-Cu^1+^	11.3%	64.5%	34.5%	7.3%	1.60	0.86
7	HNT-NH_2_ -GSH-Cu^1+^-X	42.2%	86.1%	12.9%	36.3%	7.98	1.20

a Reaction conditions: catalyst (100 mg), CO_2_ and H_2_ 1 atm, H_2_/CO_2_ molar ratio (4:1), 7 h of solar radiation (simulated), *T* = 45 °C, due to the heating of the solar lamp

Under solar light irradiation at moderate temperatures (45 °C in our case due to the heating of the used solar lamp), the H_2_ molecules dissociate into hydrogen atoms on the surface of the active sites (metal). These hydrogen atoms hydrogenate adsorbed CO_2_. In molecular metal complexes, direct catalytic oxidation of H_2_ to H^+^ is promoted, thereby enhancing CO_2_ conversion.^30,33^ For these reasons, the presence of metal active sites is fundamental. Subsequently, the generated H^+^ reacted with the adsorbed CO_2_ at the basic sites of the HNT. The presence of the NH_2_-GSH functionalization enabled efficient chelation of Cu­(I) ions, exposing these species to the sample surface and allowing them to convert CO_2_ into CO and sequentially into methane.^9,28^ Indeed, the sample without this functionalization, HNT-Cu^1+^ (Table 3, entry 2), showed low photoactivity, confirming that the chelating action of GSH is crucial for promoting an efficient activation of the photocatalytic reaction on the cuprous ion sites. This indicated that interactions among all components of the compounds were necessary for successful CO_2_ methanation. This synergistic effect between the several elements of the proposed green photocatalyst was further improved due to the presence of xanthopterin, which enhanced the harvesting of solar photons, resulting in optimal CO_2_ conversion efficiency (42%) and enhancing the CH_4_ selectivity (86%, CH_4_ formation rate 7.98 μmol/g_cat_·h) and yield (36%) (Table 3, entry 7).

Although it is very difficult to compare the obtained results with other ones investigated photocatalysts for the solar CO_2_ methanation, due to the various experimental setups and conditions reported in the literature (such as the type of used lamp, duration of irradiation, temperature, pressure, etc.), the performance of the HNT-NH_2_ -GSH-Cu^1+^-X sample is promising in terms of CH_4_ formation rate, selectivity, and yield, considering that in the same experimental conditions, the standard Cu_2_O/TiO_2_-based semiconductor oxides exhibited a similar CO_2_ conversion (45%), but favoring the CO formation 12 μmol/g_cat_·h, with a selectivity of 67%, rather than the methane formation (5 μmol/g_cat_·h CH_4_ selectivity = 33%).^54^ Indeed, with the here-examined HNT-NH_2_-GSH-Cu^1+^-X nanocatalyst, a higher CH_4_ formation (7.98 μmol/g_cat_·h) was achieved with higher selectivity (86%).

Higher CH_4_ selectivity (up to 97%) can be achieved using other Cu_2_O-based materials;^55^ however, this comes at the cost of reduced biocompatibility and sustainability compared with our approach. Indeed, in the present work, Cu­(I) ions are exclusively chelated within the hybrid sustainable composite structure, in contrast to the use of bare Cu_2_O-based semiconductors, thereby improving the overall sustainability of the process.

For the best-performing nanocatalysts (HNT-NH_2_-GSH, HNT-NH_2_-GSH-Cu^1+^, and HNT-NH_2_-GSH-Cu^1+^-X), the optical properties were evaluated by UV-DRS characterization (Figure S4). All of the nanomaterials exhibit a broad absorption band in the 250–350 nm range, attributed to the electronic transitions of GSH molecules.^56^ The presence of Cu ions led to an additional feature in the 500–600 nm range, related to the localized surface plasmon resonance of copper.^57^ This feature was more intense and red-shifted in the HNT-NH_2_-GSH-Cu^1+‑^-X sample due to the additional absorption features of the xanthopterin antenna in the same visible light range.^28^ This increased absorption in the visible range of the HNT-NH_2_-GSH-Cu^1+‑^-X nanocomposite improved the absorption and utilization of simulated solar light in the photocatalytic tests. Therefore, the combined action of the Cu ions and the antenna molecules enhanced the sample’s solar photocatalytic performance, thereby improving it (Table 3, entry 7).

For the same samples, the textural properties were determined (Figure S5, Table S1). All of the examined nanocomposites exhibited a type IV isotherm with an H_2_ hysteresis loop (Figure S5A), consistent with pores with wide bodies and narrow necks.^59^ The addition of Cu ions and of the antenna molecule did not give substantial variations of the BET surface area (≈67–68 m^2^/g, Table S1), nor in the pore size distribution (Figure S5B), with a narrow distribution centered on mesopores of 10–11 nm. As expected, the textural properties were mainly determined by the presence of halloysite, the common support of all examined samples; therefore, no substantial variations were observed among the investigated nanomaterials.

Finally, Figure 6 reports the photocatalytic reusability of the best photocatalytically active sample, HNT-NH_2_-GSH-Cu^1+‑^X, after five runs of the photocatalytic reaction (each run of 7 h of simulated solar irradiation). At the end of each run, the sample was flushed with He (30 cc/min) for 2 h to remove any carbonaceous species physiosorbed on the sample surface and to reuse the sample. The nanomaterial showed good stability, with only a slight decrease in the CO_2_ conversion after the fourth run (i.e., 28 h of simulated solar irradiation). This little reduction in performance can be reasonably attributed to the partial oxidation of Cu­(I) into Cu­(II), which are less active with respect to the cuprous ions.^28,60,61^ Despite this, the overall stability of HNT-NH_2_-GSH-Cu^1+‑^X was optimal, indicating its potential for practical use in solar photocatalytic CO_2_ conversion.

**Figure 6:**
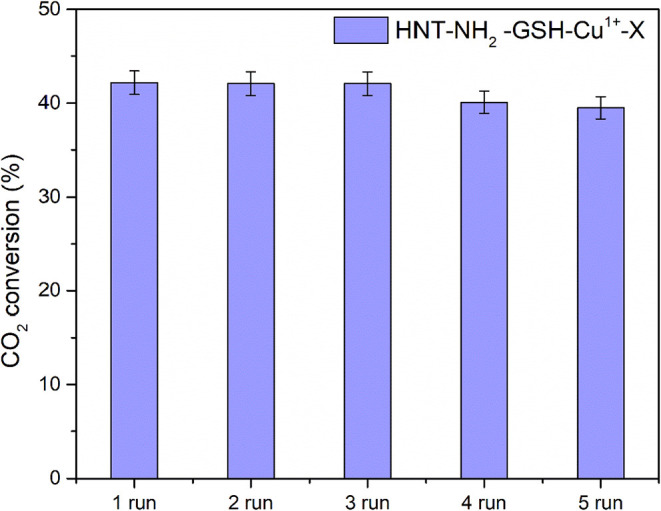
Photocatalytic runs (each run of 7 h of simulated solar irradiation) for the HNT-NH_2_ -GSH-Cu^1+–^X photocatalyst in the CO_2_ reduction reaction.

### 3.4 Sensor Activity

The sensing capabilities of bare SPCE and HNT-NH_2_-GSH-Cu^2+^/SPCE were evaluated by CV analysis upon 250 nM bisphenol A (0.1 μL of a 10 mM solution of the bisphenol A added to 4 mL of a PBS 0.1 M electrolyte solution). Figure 7 shows the anodic oxidation peak (IPox) at approximately 0.6 and 0.5 V, for the SPCE and HNT-GSH-Cu^2+^/SPCE, respectively. The observed response for HNT-NH_2_-GSH-Cu^2+^/SPCE is about three times as great as for SPCE. In addition, the shift to a lower peak potential and the increase in oxidation current imply faster electron-transfer kinetics in the HNT-NH_2_-GSH-Cu^2+^/SPCE sensor.^62^

**Figure 7:**
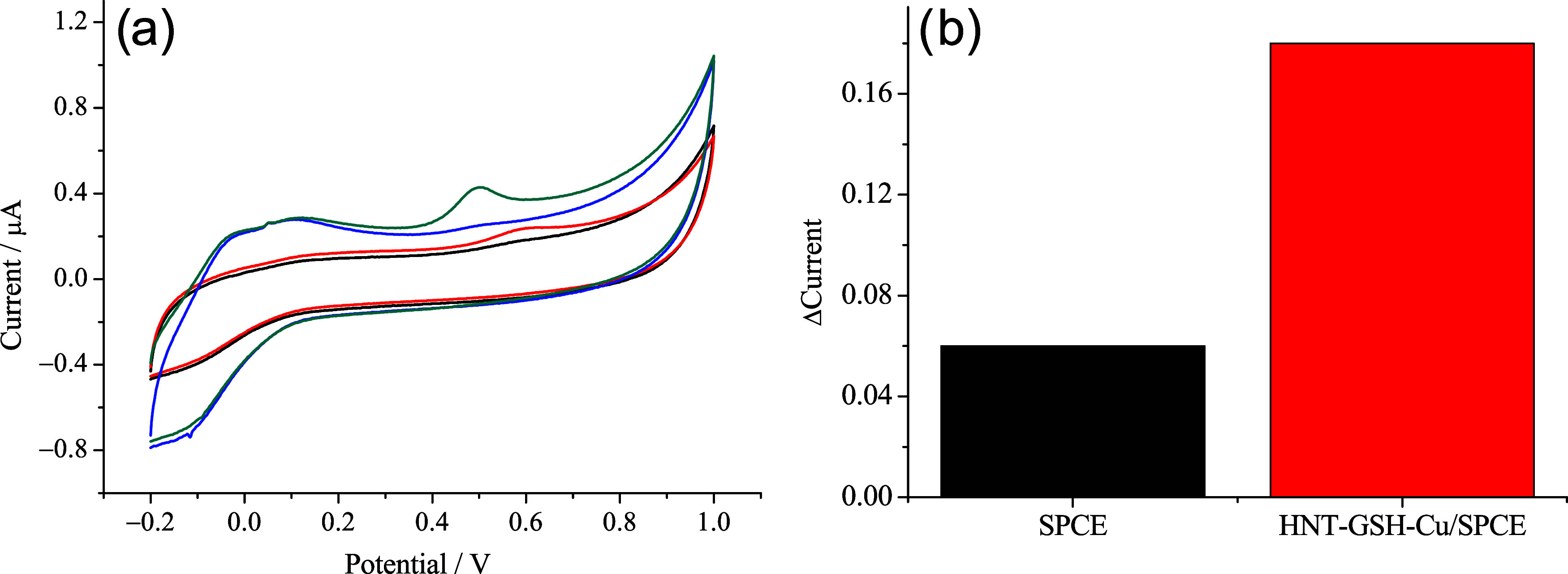
(a) Electrochemical behavior of SPCE (black and red lines), and HNT-NH_2_-GSH-Cu^2+^/SPCE (blue and green lines) in −0.2–1.0 V potential range, before and after 250 nM of bisphenol A; (b) comparison between the responses.

The detection capabilities of HNT-NH_2_-GSH-Cu^2+^/SPCE were examined by LSV and DPV measurements (Figure 8a,c) using increasing bisphenol A concentrations. Figure 8b,d shows the related calibration curves. This nanosensor exhibits a sensitivity of 5.098 μA μM^–1^ cm^–2^, and an LOD of 0.022 μM, thus providing an excellent responsiveness to bisphenol A.

**Figure 8:**
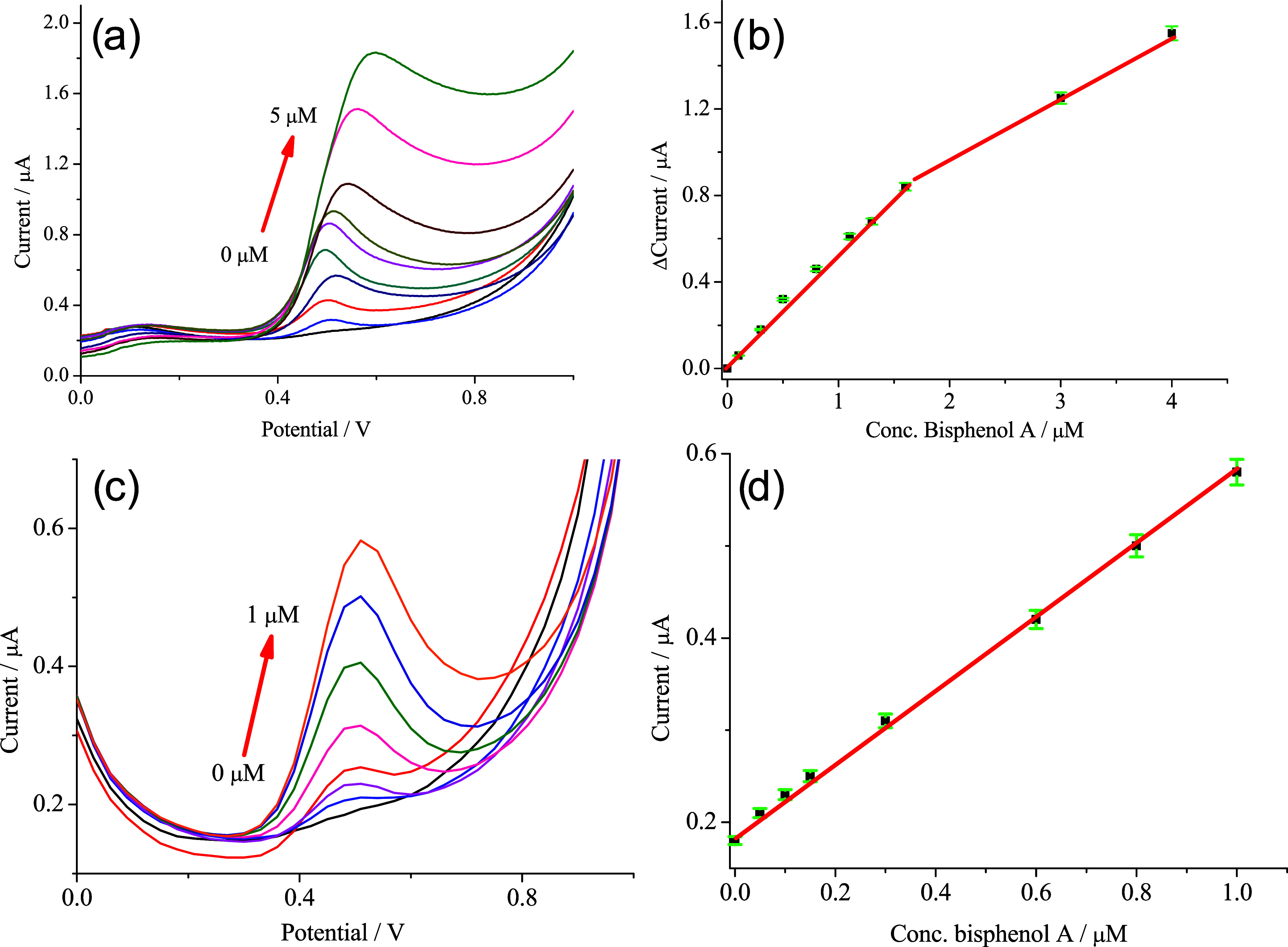
(a) LSV at different bisphenol A concentrations (0–5 μM, initial step 0.1 μM) in 0.1 M PBS (pH 7.4); (b) calibration curve for anodic peak current (*I*_pa_) vs the bisphenol A (RSD ≤ 2.1 for five repeated whole cycles) (eq *y* = *a* + *b* × *x*; *R* ^2^ = 0.99763; intercept = – 0.00339 ± 0.00531; slope = 0.63729 ± 0.001794); (c) DPV at different bisphenol A concentrations (0–1 μM, initial step 0.05 μM) in 0.1 M PBS (pH 7.4); (d) calibration curve for anodic peak current (*I*_pa_) vs the bisphenol A concentration (RSD ≤ 1.8 for five repeated whole cycles) (eq *y* = *a* + *b* × *x*; *R* ^2^ = 0.9926; intercept = 0.18528 ± 0.00288; slope = 0.42264 ± 0.001823).

To highlight the importance of our nanocomposite for the electrochemical detection of bisphenol A, we present a comparative table of nanocomposite sensors reported in the literature (Table 4). It is easy to see why our composite is an excellent candidate as a sensor for detecting a contaminant, which is a significant concern at present. Although other nanosensors with lower LODs exist, the nanosensor described here is an excellent candidate due to its ease of preparation and the sustainability of its materials and processes.

**Table 4:** Comparison of the Detection Performance of HNT-NH2-GSH-Cu2+ and the Nanocomposite Sensors Reported for Bisphenol A

electrode materials	LOD μM	reference
HNT-NH_2_-GSH-Cu^2+^	0.022	this work
GCE/H-diA(6 mg)/C60(8 mg)	0.0014	^63^
MIP/CNTs-Au NPs/BOMC	0.005	^64^
SERS	0.003	^65^
NiCo-MOF/LPC-700/SPE	0.049	^66^
Ni–Cu(PDA)MOF/CNF/GC	0.075	^67^
Au@MIP	0.052	^68^
MIPPy/GQDs	0.040	^69^
carbon felt	0.480	^70^
exfoliated graphite (EG)	0.076	^71^
[Ru(bpy)^3^]^2+^ on the ITO	0.029	^72^
MIP/Fe_3_O_4_ NPs	0.380	^73^

## 5 Conclusions

This research successfully demonstrates the creation of a highly sustainable and effective multifunctional platform for simultaneous photocatalysis and electrochemical sensing. By leveraging natural and eco-biocompatible nanomaterials such as halloysite nanotubes, glutathione, and xanthopterin, and employing green synthetic methods, we have developed a nanocomposite that not only aligns with environmental sustainability principles but also delivers exceptional performance. The synergistic interplay among the HNT scaffold, the GSH chelating agent, the copper ions, and the xanthopterin antenna is crucial, as evidenced by the high CO_2_ conversion and methane selectivity achieved under solar light. The nanomaterial’s superior performance in CH_4_ production compared to conventional photocatalysts highlights the advantages of this bioinspired approach. Additionally, the same nanosystem exhibits impressive capabilities for detecting bisphenol A, confirming its versatility and potential for dual-purpose applications. This work provides a promising blueprint for designing next-generation, environmentally friendly nanomaterials that can address global challenges in energy and environmental protection in an integrated and efficient manner. In real-world scenarios, it could simultaneously contribute to CO_2_ mitigation while providing sensitive monitoring of hazardous pollutants. Such versatility makes the nanosystems particularly attractive for industrial wastewater treatment, air quality monitoring, and sustainable energy production. Overall, the multifunctional design represents a significant step toward eco-friendly, cost-effective environmental technologies.

## Supplementary Material



## Notes

Stacked UV spectra of xanthopterin (0.025 mg/mL in DMSO) and supernatant for the preparation of HNT-NH_2_-GSH-Cu^1+^(Figure S1); Al–Kα excited XPS of HNT-NH_2_-GSH-Cu^1+^-X in the 0–1200 binding energy region (Figure S2); Al Kα excited XPS of the HNT-NH_2_-GSH-Cu^1+^-X samples in the in the: (a) C 1s binding energy region; (b) O 1s binding energy region; (c) N 1s binding energy region; (d) Al 2p binding energy region; (e) Si 2p binding energy region; (f) S 2p binding energy region (Figure S3); UV-DRS spectra of the examined samples (Figure S4); (A) N_2_ isotherms and (B) pore size distribution of the examined samples (Figure S5); textural properties of the examined samples (Table S1) (PDF)

## References

[1] Liu W.-J., Jiang H., Yu H.-Q. (2015). Development
of biochar-based functional
materials: toward a sustainable platform carbon material. Chem. Rev..

[2] Moura B., Rosero-Romo J., Monteiro H., Alberto A., Laranjeira J., Martin-Iglesias S., Silvan U., Lanceros-Mendez S., Salazar D., Martins C. (2024). Addressing safety and sustainability
issues in the development of nano-enabled MULTI-FUNctional materials
for metal additive manufacturing. Sustainable
Mater. Technol..

[3] Irimia-Vladu M. (2014). Green”
electronics: biodegradable and biocompatible materials and devices
for a sustainable future. Chem. Soc. Rev..

[4] Han W. B., Yang S. M., Rajaram K., Hwang S. W. (2022). Materials and Fabrication
Strategies for Biocompatible and Biodegradable Conductive Polymer
Composites toward Bio-Integrated Electronic Systems. Adv. Sustainable Syst..

[5] Patamia V., Fiorenza R., Zagni C., Agustin-Salazar S., Scirè S., Floresta G., Rescifina A. (2024). TiO2/Loofah–Halloysite
Bio–Hybrid Composites as Efficient Systems for VOCs Removal. Chem. - Eur. J..

[6] Saccullo E., Patamia V., Bifarella A., Ferlazzo A., Fiorenza R., Spitaleri L., Sfuncia G., Nicotra G., Zagni C., Iapichino M. T. A. (2025). Conversion of VOC-derived CO2 into sustainable
products with a natural magnetic alginate composite. Int. J. Biol. Macromol..

[7] Patamia V., Saccullo E., Magaletti F., Fuochi V., Furnari S., Fiorenza R., Furneri P. M., Barbera V., Floresta G., Rescifina A. (2024). Nature-inspired
innovation: Alginic-kojic acid material
for sustainable antibacterial and carbon dioxide fixation. Int. J. Biol. Macromol..

[8] Massaro M., Colletti C., Lazzara G., Milioto S., Noto R., Riela S. (2017). Halloysite nanotubes
as support for metal-based catalysts. J. Mater.
Chem. A.

[9] Saccullo E., Patamia V., Magaletti F., Dativo G., Camarda M., Fiorenza R., Barbera V., Floresta G., Rescifina A. (2024). Halloysite-kojic
acid conjugate: A sustainable material for the photocatalytic CO2
reduction and fixation for cyclic carbonates production. J. CO_2_ Util..

[10] Failla M., Ferlazzo A., Abbate V., Neri G., Saccullo E., Gulino A., Rescifina A., Patamia V., Floresta G. (2024). THP as a sensor
for the electrochemical detection of H2O2. Bioorg.
Chem..

[11] Heras J. Y., Rodriguez S. D., Negri R. M., Battaglini F. (2010). Chelating
electrodes as taste sensor for the trace assessment of metal ions. Sens. Actuators, B.

[12] Ferlazzo A., Chelly M., Gulino A., Neri G. (2025). Biosensing of Urea
with a Functionalized Gold Electrode for Health and Food Monitoring. J. Agric. Food Chem..

[13] Jia F., Liu Q., Chen Z., Wei W. (2019). Colorimetric determination of nine
metal ions based on the de-aggregation of papain-functionalized gold
nanoparticles and using three chelating agents. Microchim. Acta.

[14] Lauriane N., Najih R., Chtaini A. (2014). Electrochemical
sensor of heavy metals
based on chelating compounds. Pharm. Anal. Acta.

[15] Patamia V., Fiorenza R., Brullo I., Marsala M. Z., Balsamo S. A., Distefano A., Furneri P. M., Barbera V., Scirè S., Rescifina A. (2022). A sustainable porous composite material
based on loofah-halloysite
for gas adsorption and drug delivery. Mater.
Chem. Front..

[16] Sadjadi S. (2020). Halloysite-based
hybrids/composites in catalysis. Appl. Clay
Sci..

[17] Patamia V., Zagni C., Fiorenza R., Fuochi V., Dattilo S., Riccobene P. M., Furneri P. M., Floresta G., Rescifina A. (2023). Total Bio-Based
Material for Drug Delivery and Iron Chelation to Fight Cancer through
Antimicrobial Activity. Nanomaterials.

[18] Ferlazzo A., Iapichino M. T. A., Calabrese G., D’Accurso G., Fiorenza R., Pistarà V., Gulino A., Rescifina A., Patamia V., Floresta G. (2025). Nanosensors
Made of Halloysite and
Kojic Acid Metal Complexes for Dopamine Detection. ACS Appl. Nano Mater..

[19] Goda E. S., Gab-Allah M., Singu B. S., Yoon K. R. (2019). Halloysite nanotubes
based electrochemical sensors: A review. Microchem.
J..

[20] Jeamjumnunja K., Cheycharoen O., Phongzitthiganna N., Hannongbua S., Prasittichai C. (2021). Surface-modified halloysite nanotubes as electrochemical
CO2 sensors. ACS Appl. Nano Mater..

[21] Meng S.-L., Li J.-H., Ye C., Yin Y.-L., Zhang X.-L., Zhang C., Li X.-B., Tung C.-H., Wu L.-Z. (2024). Concurrent
Ammonia Synthesis and Alcohol Oxidation Boosted by Glutathione-Capped
Quantum Dots under Visible Light. Adv. Mater..

[22] Wang W., Wang T., Chen S., Lv Y., Salmon L., Espuche B., Moya S., Morozova O., Yun Y., Di Silvio D. (2024). Cu (I)-Glutathione Assembly Supported on ZIF-8 as Robust
and Efficient Catalyst for Mild CO2 Conversions. Angew. Chem..

[23] Cui X., Wang J., Liu B., Ling S., Long R., Xiong Y. (2018). Turning Au Nanoclusters
Catalytically Active for Visible-Light-Driven
CO2 Reduction through Bridging Ligands. J. Am.
Chem. Soc..

[24] Bagherpour S., Pérez-García L. (2024). Recent advances on nanomaterial-based
glutathione sensors. J. Mater. Chem. B.

[25] Han Q., Shen X., Zhu W., Zhu C., Zhou X., Jiang H. (2016). Magnetic sensing film
based on Fe3O4@ Au-GSH molecularly imprinted
polymers for the electrochemical detection of estradiol. Biosens. Bioelectron..

[26] Ai L., Liu Z., Zhang X., Wang L., Jia D., Guo N., Zha M., Tan C. (2025). Engineering cycling of Cu2+/Cu+ pairs
in Bi2WO6 nanoflowers
for boosting photocatalytic CO2 reduction. J.
Colloid Interface Sci..

[27] Mao Y., Zhang M., Zhai G., Si S., Liu D., Song K., Liu Y., Wang Z., Zheng Z., Wang P. (2024). Asymmetric Cu (I)–W Dual-Atomic Sites Enable
C–C Coupling for Selective Photocatalytic CO2 Reduction to
C2H4. Adv. Sci..

[28] Saccullo E., Dativo G., Lombardo R., Iapichino M. T. A., Fiorenza R., Patamia V., Floresta G. (2025). Natural antenna molecule
in halloysite-kojic acid composite for the solar photocatalytic CO2
methanation. Mater. Today Chem..

[29] Xiong J., Wang Y., Xue Q., Wu X. (2011). Synthesis
of highly
stable dispersions of nanosized copper particles using L-ascorbic
acid. Green Chem..

[30] Chen Y., Zhang Y., Fan G., Song L., Jia G., Huang H., Ouyang S., Ye J., Li Z., Zou Z. (2021). Cooperative catalysis coupling photo-/photothermal
effect to drive
Sabatier reaction with unprecedented conversion and selectivity. Joule.

[31] Long R., Li Y., Liu Y., Chen S., Zheng X., Gao C., He C., Chen N., Qi Z., Song L. (2017). Isolation
of Cu atoms in Pd lattice: forming highly selective sites for photocatalytic
conversion of CO2 to CH4. J. Am. Chem. Soc..

[32] Fiorenza R., Bellardita M., Balsamo S. A., Spitaleri L., Gulino A., Condorelli M., D’Urso L., Scire S., Palmisano L. (2022). A solar photothermocatalytic approach
for the CO2 conversion: Investigation of different synergisms on CoO-CuO/brookite
TiO2-CeO2 catalysts. Chem. Eng. J..

[33] Zhang P., Sui X., Wang Y., Wang Z., Zhao J., Wen N., Chen H., Huang H., Zhang Z., Yuan R. (2023). Surface
Ru–H bipyridine complexes-grafted TiO2 nanohybrids
for efficient photocatalytic CO2 methanation. J. Am. Chem. Soc..

[34] Ferlazzo A., Gulino A., Neri G. (2024). Scandia-doped
zirconia for the electrochemical
detection of hazardous dihydroxybenzene (DHB) isomers in water. Environ. Sci.: Adv..

[35] Maugeri L., Fangano G., Ferlazzo A., Forte G., Gulino A., Petralia S. (2024). A DNA biosensor integrating surface hybridization,
thermo-responsive coating, laminar-flow technology and localized photothermal
effect for efficient electrochemical detection of nucleic acids. Sens. Diagn..

[36] Abid K., Ferlazzo A., Neri G. (2024). Graphene quantum dots (GQDs)-modified
screen-printed electrode for the determination of cannabidiol (CBD)
in hemp seeds flour. FlatChem.

[37] Briggs, D. ; Grant, J. Surface Analysis by Auger and X-Ray Photoelectron Spectroscopy; IMP: Chichester, UK, 2003.

[38] Gulino A. (2013). Structural
and electronic characterization of self-assembled molecular nanoarchitectures
by X-ray photoelectron spectroscopy. Anal. Bioanal.
Chem..

[39] Greczynski G., Hultman L. (2020). Compromising science by ignorant instrument calibration—need
to revisit half a century of published XPS data. Angew. Chem., Int. Ed..

[40] Dastidar R. G., Kim M. S., Zhou P., Luo Z., Shi C., Barnett K. J., McClelland D. J., Chen E. Y.-X., Van
Lehn R. C., Huber G. W. (2022). Catalytic production of tetrahydropyran
(THP): a biomass-derived, economically competitive solvent with demonstrated
use in plastic dissolution. Green Chem..

[41] Shi Y., Sun K., Shan J., Li H., Gao J., Chen Z., Sun C., Shuai Y., Wang Z. (2022). Selective CO2 Electromethanation
on Surface-Modified Cu Catalyst by Local Microenvironment Modulation. ACS Catal..

[42] Wang W., Wang T., Chen S., Lv Y., Salmon L., Espuche B., Moya S., Morozova O., Yun Y., Di Silvio D., Daro N., Berlande M., Hapiot P., Pozzo J.-L., Yu H., Hamon J.-R., Astruc D. (2024). Cu­(I)-Glutathione
Assembly Supported on ZIF-8 as Robust and Efficient Catalyst for Mild
CO2 Conversions. Angew. Chem., Int. Ed..

[43] Plotkin M., Volynchik S., Ermakov N. Y., Benyamini A., Boiko Y., Bergman D. J., Ishay J. S. (2009). Xanthopterin in
the oriental hornet (Vespa orientalis): light absorbance is increased
with maturation of yellow pigment granules. Photochem. Photobiol..

[44] Peixoto A. F., Fernandes A. C., Pereira C., Pires J., Freire C. (2016). Physicochemical
characterization of organosilylated halloysite clay nanotubes. Microporous Mesoporous Mater..

[45] Vergaro V., Abdullayev E., Lvov Y. M., Zeitoun A., Cingolani R., Rinaldi R., Leporatti S. (2010). Cytocompatibility and uptake of halloysite
clay nanotubes. Biomacromolecules.

[46] Wu D., Delair T. (2015). Stabilization of chitosan/hyaluronan
colloidal polyelectrolyte
complexes in physiological conditions. Carbohydr.
Polym..

[47] Gulino A., Lupo F., Condorelli G. G., Fragalà M. E., Amato M. E., Scarlata G. (2008). Reversible photoswitching of stimuli-responsive
Si (100) surfaces engineered with an assembled 1-cyano-1-phenyl-2-[4′-(10-undecenyloxy)
phenyl]-ethylene monolayer. J. Mater. Chem..

[48] Gulino A., Condorelli G. G., Mineo P., Fragalà I. (2005). An x-ray photoelectron
spectra and atomic force microscopy characterization of silicasubstrates
engineered with a covalently assembled siloxane monolayer. Nanotechnology.

[49] Cristaldi D. A., Fortuna C. G., Gulino A. (2013). A photoelectron spectroscopy
study
of lava stones. Anal. Methods.

[50] Florio F., Ferlazzo A., Bonforte S., Nicotra G., Neri G., Pinkas I., van der
Boom M. E., Gulino A. (2025). Unveiling the sensing
ability of new MoS 2 nanoparticles: from fundamental insights into
practical applications for nitrites. J. Mater.
Chem. C.

[51] Weser, U. Redox reactions of sulphur-containing amino-acid residues in proteins and metalloproteins, an XPS study. In Cation Ordering and Electron Transfer, Structure and Bonding; Springer, 2007; pp 145–160 10.1007/bfb0111194.

[52] Ferlazzo A., Bonforte S., Florio F., Petralia S., Sorace L., Muzzi B., Caneschi A., Gulino A. (2024). Photochemical
eco-friendly
synthesis of photothermal and emissive copper nanoclusters in water:
towards sustainable nanomaterials. Mater. Adv..

[53] Kaminker R., De Hatten X., Lahav M., Lupo F., Gulino A., Evmenenko G., Dutta P., Browne C., Nitschke J. R., Van Der
Boom M. E. (2013). Assembly of surface-confined homochiral helicates:
chiral discrimination of DOPA and unidirectional charge transfer. J. Am. Chem. Soc..

[54] Fiorenza R., Contarino C., Spano V., Iapichino M. T. A., Balsamo S. A. (2023). Photothermo-catalytic strategies for the CO2 valorisation
using TiO2-based composites. Catal. Today.

[55] Deng Y., Wan C., Li C., Wang Y., Mu X., Liu W., Huang Y., Wong P. K., Ye L. (2022). Synergy effect between
facet and zero-valent copper for selectivity photocatalytic methane
formation from CO2. ACS Catal..

[56] Singh G., Dogra S. D., Kaur S., Tripathi S., Prakash S., Rai B., Saini G. (2015). Structure
and vibrations of glutathione studied by
vibrational spectroscopy and density functional theory. Spectrochim. Acta, Part A.

[57] Popok V. N., Novikov S. M., Lebedinskij Y. Y., Markeev A. M., Andreev A. A., Trunkin I. N., Arsenin A. V., Volkov V. S. (2021). Gas-aggregated copper
nanoparticles with long-term plasmon resonance stability. Plasmonics.

[59] Thommes M., Kaneko K., Neimark A. V., Olivier J. P., Rodriguez-Reinoso F., Rouquerol J., Sing K. S. W. (2015). Physisorption
of gases, with special
reference to the evaluation of surface area and pore size distribution
(IUPAC Technical Report). Pure Appl. Chem..

[60] Fiorenza R., Bellardita M., Balsamo S. A., Gulino A., Condorelli M., Compagnini G., Scirè S., Palmisano L. (2023). A solar photothermo-catalytic
combined process for the VOCs combustion and the subsequent CO2 valorization
using noble metal-free catalysts. Catal. Today.

[61] Celaya C. A., Delesma C., Torres-Arellano S., Sebastian P., Muniz J. (2021). Understanding CO2 conversion into
hydrocarbons via a photoreductive
process supported on the Cu2O (1 0 0),(1 1 0) and (1 1 1) surface
facets: A first principles study. Fuel.

[62] Ferlazzo A., Espro C., Iannazzo D., Bonavita A., Neri G. (2023). Yttria-zirconia
electrochemical sensor for the detection of tyrosine. Mater. Today Commun..

[63] Tchieno F. M. M., Dmitrieva E., Boye S., Schiemenz S., Kluge R. (2023). Diamine@halloysite/C60 composite-based Bisphenol A electrochemical
sensor. J. Electroanal. Chem..

[64] Hu X., Feng Y., Wang H., Zhao F., Zeng B. (2018). A novel bisphenol
A electrochemical sensor based on a molecularly imprinted polymer/carbon
nanotubes-Au nanoparticles/boron-doped ordered mesoporous carbon composite. Anal. Methods.

[65] Li S., He D., Li S., Chen R., Peng Y., Li S., Han D., Wang Y., Qin K., Ren S., Chen P., Gao Z. (2022). Magnetic Halloysite Nanotube-Based SERS Biosensor Enhanced with Au@Ag
Core–Shell Nanotags for Bisphenol A Determination. Biosensors.

[66] Wang Y., Liu Z., Zhao Z., Fei M., Xie Y., Guo H., Zhao P., Fei J. (2024). Modulation of metal centers of MOF
in-situ grown on lignin-derived carbon to enhance adsorption capacity
and electrochemical sensing performance for bisphenol A. Chem. Eng. J..

[67] Dey B., Ahmad M. W., Al-Shannaq R., Al-Humaidi J. Y., Hossain S. K. S., Patra C. N., Althomali R. H., Rahman M. M., Choudhury A. (2024). Non-Enzymatic Electrochemical Sensing
of Bisphenol A in Drinking Water and Milk Using Bimetallic Nickel-Copper
Metal–Organic Framework. J. Anal. Test..

[68] Han E., Pan Y., Li L., Cai J. (2023). Bisphenol A detection based on nano
gold-doped molecular imprinting electrochemical sensor with enhanced
sensitivity. Food Chem..

[69] Tan F., Cong L., Li X., Zhao Q., Zhao H., Quan X., Chen J. (2016). An electrochemical
sensor based on
molecularly imprinted polypyrrole/graphene quantum dots composite
for detection of bisphenol A in water samples. Sens. Actuators, B.

[70] Kim M., Song Y. E., Xiong J.-Q., Kim K.-Y., Jang M., Jeon B.-H., Kim J. R. (2021). Electrochemical detection and simultaneous
removal of endocrine disruptor, bisphenol A using a carbon felt electrode. J. Electroanal. Chem..

[71] Ndlovu T., Arotiba O. A., Sampath S., Krause R. W., Mamba B. B. (2012). An Exfoliated
Graphite-Based Bisphenol A Electrochemical Sensor. Sensors.

[72] Li Q., Li H., Du G.-F., Xu Z.-H. (2010). Electrochemical detection of bisphenol
A mediated by [Ru­(bpy)­3]­2+ on an ITO electrode. J. Hazard. Mater..

[73] Kaassamani R. A., Sawan S., Jaffrezic-Renault N., Maalouf R. (2025). A novel electrochemical
sensor based on iron oxide nanoparticles coated with molecularly imprinted
polymers for Bisphenol A detection. Microchem.
J..

