# Supplementary material for: Multifunctional Halloysite-Glutathione Nanocomposite for Solar CO2 Conversion and Pollutant Sensing
Source: ACS Appl Nano Mater. 2026 Feb 14;9(8):3941–52. doi: 10.1021/acsanm.5c05781 (PMC12956140; doi:10.1021/acsanm.5c05781)
Supplement: Supplementary file 1 [file an5c05781_si_001.pdf]

Supporting Information

Multifunctional Halloysite-Glutathione Nanocomposite for Solar CO_2 Conversion and Pollutant Sensing

Erika Saccullo [a,b], Angelo Ferlazzo [c], Giusy Dativo [c], Roberto Fiorenza [c], Giulia Sambataro [a], Elena Bruno [d], Antonino Gulino [c], Antonio Rescifina [a], Vincenzo Patamia [a,*], Giuseppe Floresta [a,*]

[a] *Department of Drug and Health Sciences, University of Catania, Viale Andrea Doria 6, 95125 Catania, Italy*

[b] *Department of Biomedical and Biotechnological Sciences (Biometec), University of Catania, Via Santa Sofia 97, 95123 Catania, Italy.*

[c] *Department of Chemical Sciences, University of Catania, Viale Andrea Doria 6, 95125 Catania, Italy.*

[d] *Department of Physics and Astronomy "Ettore Majorana", University of Catania, via S. Sofia 64, 95123 Catania, Italy, and CNR-IMM, Via Santa Sofia 64, 95123 Catania, Italy.*

[*] Corresponding author.

E-mail address: vincenzo.patamia@unict.it (V. Patamia), giuseppe.floresta@unict.it (G. Floresta)

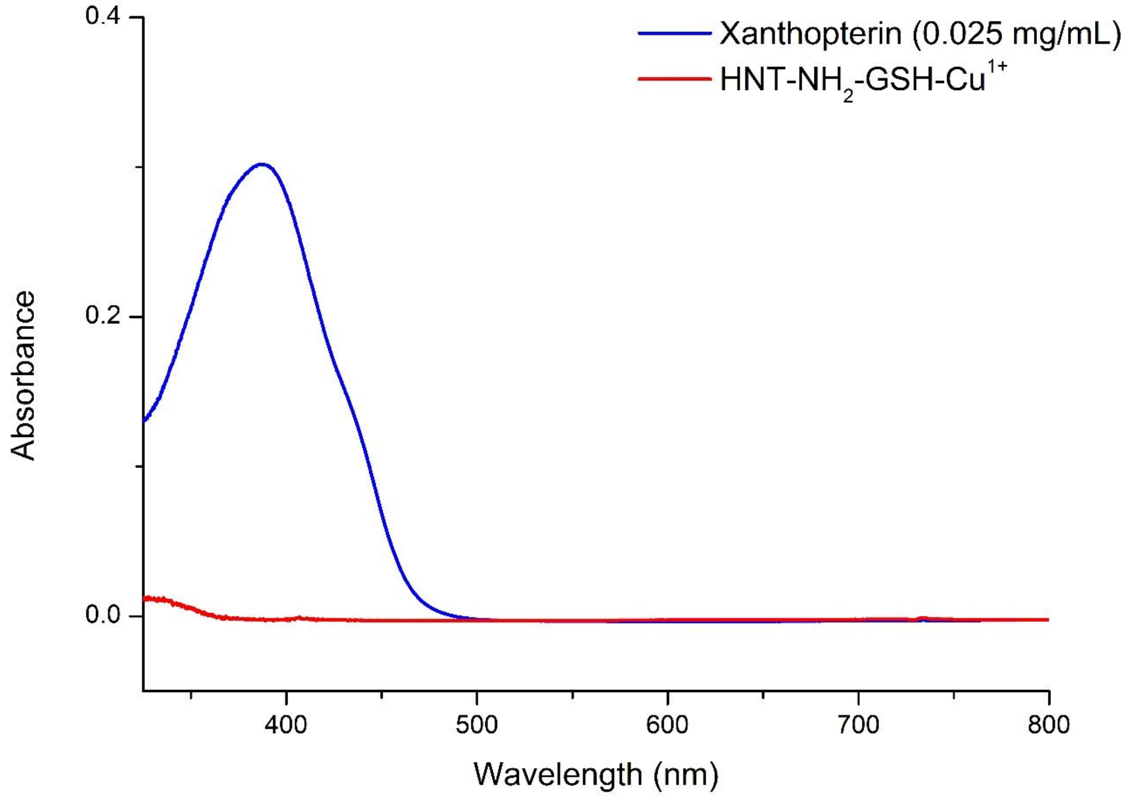

Figure S1. Stacked UV spectra of xanthopterin (0.025 mg/mL in DMSO) (blue line) and supernatant for the preparation of HNT-NH_2-GSH-Cu^{1+} (red line).

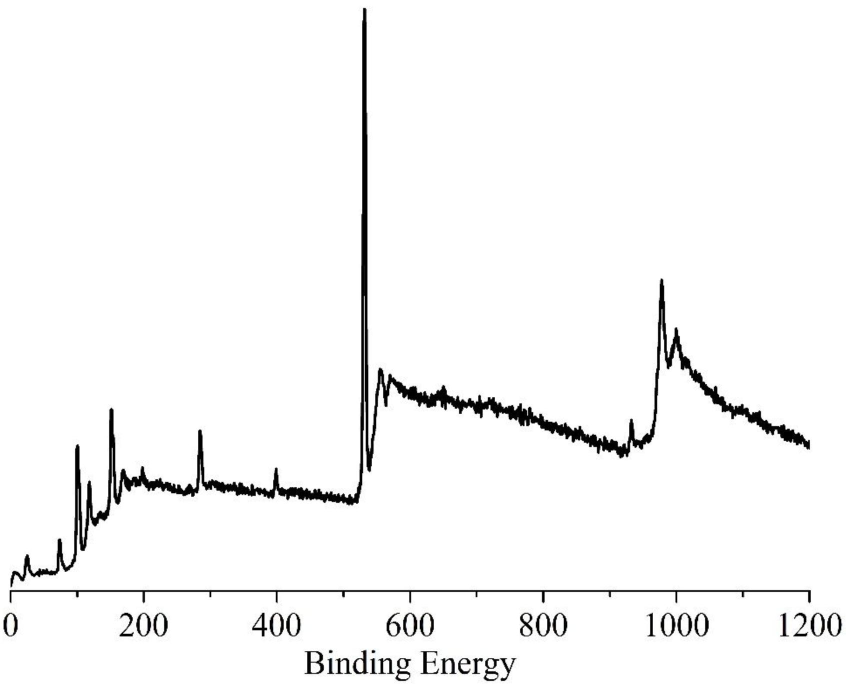

Figure S2. Al-Kα excited XPS of HNT-NH_2-GSH-Cu^{1+}-X in the 0–1200 binding energy region: survey spectrum.

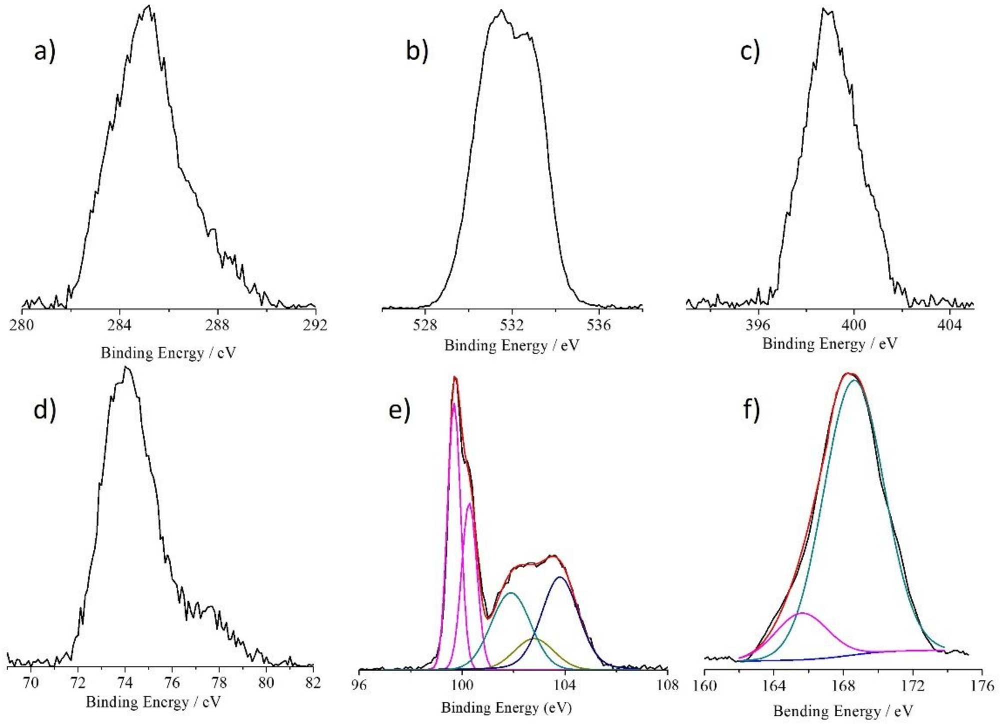

Figure S3. Al Kα excited XPS of the HNT-NH2-GSH-Cu^{1+}-X samples in the in the: a) C 1s binding energy region; b) O 1s binding energy region; c) N 1s binding energy region; d) Al 2p binding energy regione; e) Si 2p binding

energy region: the magenta, dark cyan, dark yellow, and navy lines refer to the 99.7–100.3 (relating to the $2p_{3/2,1/2}$ spin-orbit components of the Si^0 in the sample holder.), 101.9, 102.8, and 103.8 eV Gaussians components, respectively; f) S 2p binding energy region: the magenta and dark cyan lines refer to the 165.6, and 168.6 eV Gaussians components, respectively. The blue line represents the background, and the red line superimposed to the experimental black profile refers to the sum of the Gaussian components.

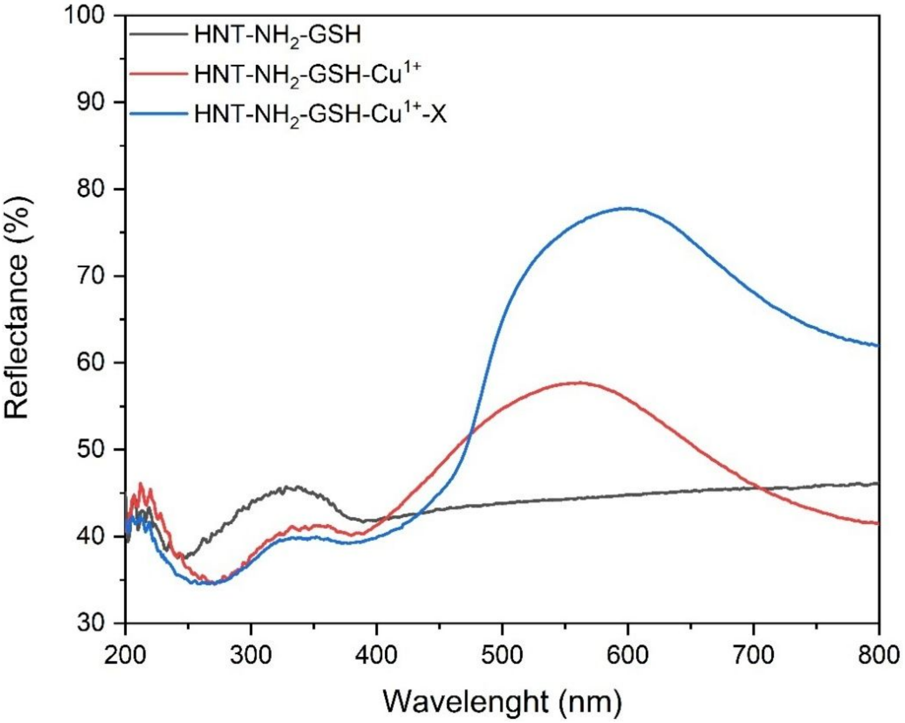

Figure S4. UV-DRS spectra of the examined samples.

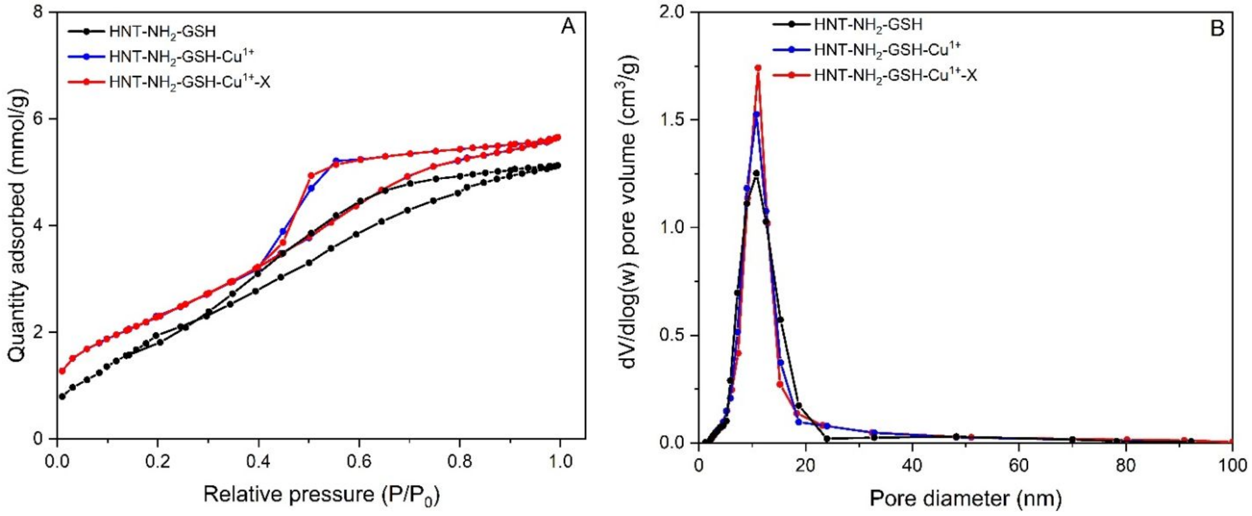

Figure S5. (A) N_2 isotherms and (B) pore size distribution of the examined samples.

Table S1. Textural properties of the examined samples.

Sample	BET surface area (m^2/g)	Mean pore diameter (nm)	Pore volume (cm^3/g)
HNT-NH_2-GSH	67.3	10.8	0.28
HNT-NH_2-GSH-Cu^{1+}	67.6	10.9	0.28
HNT-NH_2-GSH-Cu^{1+}-X	67.8	11.1	0.29
